# Identifying the snake: First scoping review on practices of communities and healthcare providers confronted with snakebite across the world

**DOI:** 10.1371/journal.pone.0229989

**Published:** 2020-03-05

**Authors:** Isabelle Bolon, Andrew M. Durso, Sara Botero Mesa, Nicolas Ray, Gabriel Alcoba, François Chappuis, Rafael Ruiz de Castañeda

**Affiliations:** 1 Institute of Global Health, Faculty of Medicine, University of Geneva, Geneva, Switzerland; 2 Institute for Environmental Sciences, University of Geneva, Geneva, Switzerland; 3 Médecins Sans Frontières, Geneva, Switzerland; 4 Division of Tropical and Humanitarian Medicine, University Hospitals of Geneva, Geneva, Switzerland; Public Health Foundation of India, INDIA

## Abstract

**Background:**

Snakebite envenoming is a major global health problem that kills or disables half a million people in the world’s poorest countries. Biting snake identification is key to understanding snakebite eco-epidemiology and optimizing its clinical management. The role of snakebite victims and healthcare providers in biting snake identification has not been studied globally.

**Objective:**

This scoping review aims to identify and characterize the practices in biting snake identification across the globe.

**Methods:**

Epidemiological studies of snakebite in humans that provide information on biting snake identification were systematically searched in *Web of Science* and *Pubmed* from inception to 2^nd^ February 2019. This search was further extended by snowball search, hand searching literature reviews, and using Google Scholar. Two independent reviewers screened publications and charted the data.

**Results:**

We analysed 150 publications reporting 33,827 snakebite cases across 35 countries. On average 70% of victims/bystanders spotted the snake responsible for the bite and 38% captured/killed it and brought it to the healthcare facility. This practice occurred in 30 countries with both fast-moving, active-foraging as well as more secretive snake species. Methods for identifying biting snakes included snake body examination, victim/bystander biting snake description, interpretation of clinical features, and laboratory tests. In nine publications, a picture of the biting snake was taken and examined by snake experts. Snakes were identified at the species/genus level in only 18,065/33,827 (53%) snakebite cases. 106 misidentifications led to inadequate victim management. The 8,885 biting snakes captured and identified were from 149 species including 71 (48%) non-venomous species.

**Conclusion:**

Snakebite victims and healthcare providers can play a central role in biting snake identification and novel approaches (e.g. photographing the snake, crowdsourcing) could help increase biting snake taxonomy collection to better understand snake ecology and snakebite epidemiology and ultimately improve snakebite management.

## Introduction

An estimated 5.4 million snake bites occur globally every year. About half of these cause snakebite envenoming (SBE), killing 81,000–138,000 people and disabling 400,000 more in the poorest regions [1, 2]. In May 2019, the World Health Organization (WHO) launched a road map to halve these deaths and disabilities by 2030, particularly focusing on the development of antivenoms and on their adequate distribution in the most affected countries [3, 4]. For this, understanding what type of snakes and associated snake bites occur where is crucial, yet this depends first on the taxonomic identification (identification hereafter; i.e. family, genus and species) of these snakes [5–7]. At the clinical level, the identity of the biting snake (BSN) can help healthcare providers anticipate victims’ syndromes and support decision making when treating the patient (i.e. whether or not to administer antivenom or which type of antivenom) [8, 9]. This decision is important because not only are antivenoms effective against a limited number of venomous snakes but they have potentially lethal side effects such as fatal allergic reactions and should not be used to treat bites from non-venomous snakes. This is especially important in view of the scarcity and high costs of antivenom vials in many countries.

However, identifying the BSN is challenging due to the high diversity of snake species [10] in snakebite endemic countries (e.g. 310 snake species in India), and the limited herpetological knowledge of communities and healthcare providers confronted with snakebite. In rural areas, traditional healers are often the first to be consulted for snakebite at the community level [11] and they could play a role in BSN identification, yet this remains to be assessed. Different behaviours and practices exist across the world depending on the development of health systems, local culture and perception of snakes and snakebite, and snake diversity. There is no standardized protocol for identifying BSNs and recommended practices are often specific to certain regions. For instance, the WHO’s Regional Office for South-East Asia (2016) [12] recommends experts identify the BSN based on a photo (i.e. taken with a mobile phone) or the animal’s body, when killed by the victim or bystanders and brought to the health facility.

To our knowledge, no review has systematically explored the literature to identify and synthesize the nature and extent of information available on the practices and challenges in BSN identification across the world. The objective of this scoping review is to provide a global and comprehensive description of the diversity of behaviours and practices of communities and healthcare providers, and the implications of these, when confronted with snakebite and the need to identify BSNs. This review also assesses the capabilities of these communities and healthcare providers to identify the BSN, and the frequency and consequences of misidentifications. Finally, it addresses aspects related to the BSNs, including their diversity and behaviour, and how this may influence their identification.

## Materials and methods

We followed the scoping review methodology proposed by Arksey & O’Malley (2005) [13], Levac et al. (2010) [14], and Tricco et al. (2018) [15] (S1 File).

### Eligibility criteria

Original epidemiological studies and clinical case report/series reporting snakebite cases in humans caused by wild snakes and including information on their taxonomic identification were considered. Studies from all geographical areas and publication dates were eligible.

### Search strategy

*Web of Science* and *Pubmed* were searched from inception to February 2, 2019 using the following key words: snakebite, snake bite, snake envenoming, snake envenomation, case, victim, event, patient, biting snake, culprit, offending snake, species, identif*, misidentif*, unidentif*, identity, and mistaking (see S1 Table for full search strategy). In addition to the database searches, we used extensive secondary search techniques, such as snowball search, hand searching literature reviews, and performing key word searches in Google Scholar. This secondary search was conducted in English, French, and Spanish by three of the authors (IB, SBM, AMD). With *PubMed*, we accessed primarily publications in the field of medicine and life sciences, while with *Web of Science* and Google Scholar we covered most scientific fields.

### Publication selection

Searched publications were merged using citation software EndNote X7 and duplicates were removed. IB, an expert in the domain of snakebite, screened all the titles and abstracts and excluded those publications that did not match eligibility criteria. Eligible publications were read by IB and SBM, and their relevance was further assessed, particularly focusing on information pertaining to the BSN (i.e. number of snakes identified and methods used). A final set of publications was produced by discussion and consensus between IB and SBM. No quality assessment of the publications selected was made.

### Charting the data

IB and SBM charted the data from selected publications independently. Their results were compared and discrepancies resolved through discussion and consensus. The variables extracted were: publication identifiers (authors, journal, year of publication, language); study characteristics (study design, country, setting, sample size); number of BSNs identified; number of victims that saw the BSN; number of BSNs captured or killed; taxonomic granularity of BSN identification (family, genus, or species); the way the BSNs were identified. We cross-referenced all common and scientific names of snakes reported in the publications with the Reptile Database [10] and kept track of changes in taxonomy where relevant. One of us (AMD) searched the literature for information on the activity time (diurnal/nocturnal/both), foraging strategy (active/passive/both), and general habitat (terrestrial, aquatic, arboreal, fossorial) of each species. Some species lacked species-specific information and their behaviour was extrapolated from those of congeners. We also gathered data on the number of snake species per country from the Reptile Database [10] and coded these as medically-important venomous snakes (MIVS) or not following the WHO. We used the pcor.test function in package ppcor (v. 1.1) in R (v. 3.5.1) to estimate partial correlation coefficients among the number of BSN species per country, the total number of snake species in that country, the total number of snake bites reported in publications from that country, and the MIVS status of the snake species.

## Results

### Selection and characteristics of publications

**Fig 1:**
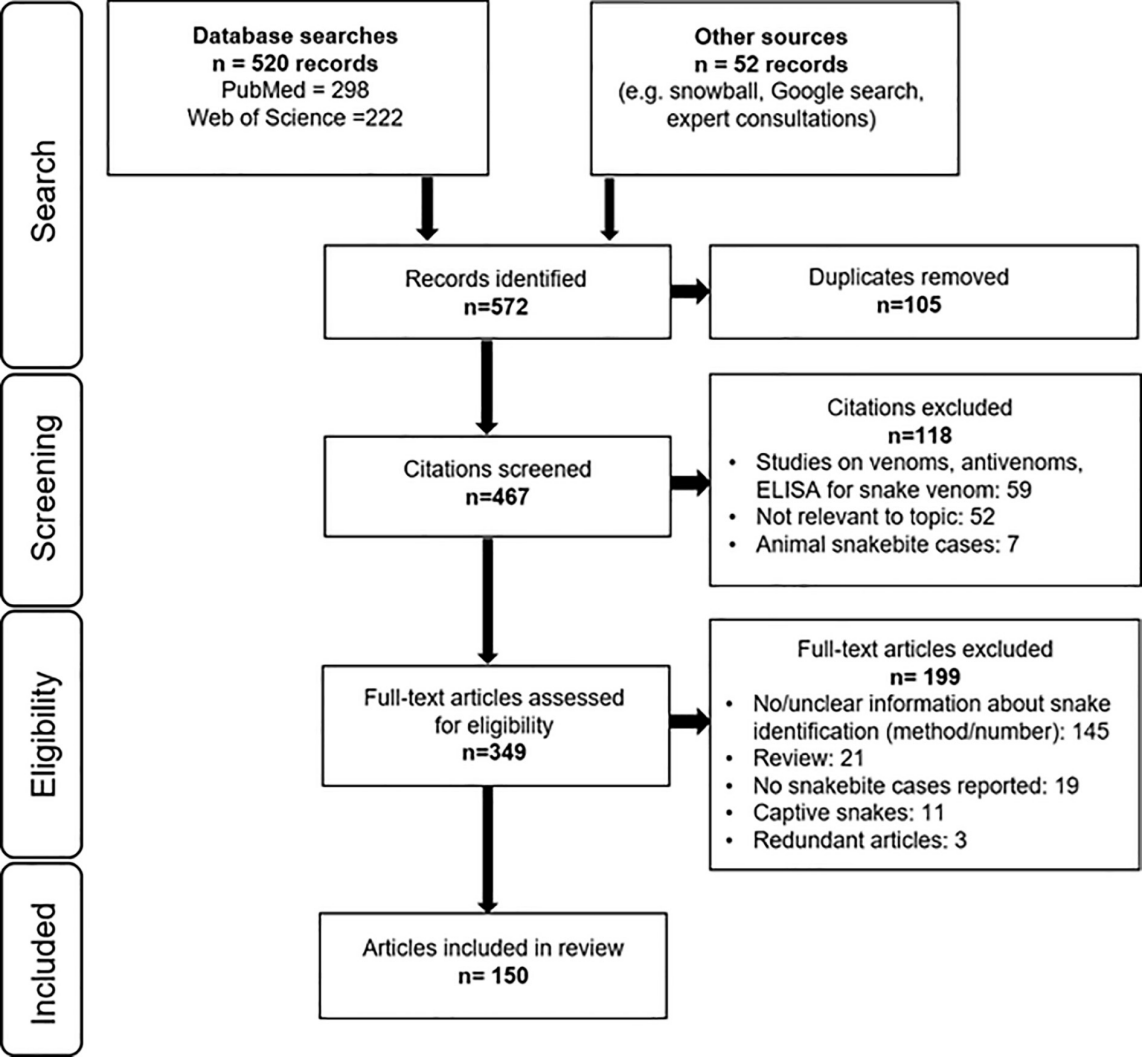
PRISMA flow diagram.

A total of 467 unique publications resulted from the initial search, and 150 of these were included in the review (Fig 1, S2 Table). These publications covered 35 countries on all continents (Table 1) and were mostly retrospective or prospective hospital-based epidemiological studies (76%), conducted in Asia (50%), published after 2000 (77%) in English (94%). In total, 33,827 snakebite cases were reported in these studies and the median number of cases per study was 90–91 (range 1–3,411).

**Table 1:** General characteristics of included publications (number of publications = 150).

Category Count (%)		
Type of study	Hospital-based retrospective study	58 (38.7)
	Hospital-based prospective study	56 (37.3)
	Case report or case-series	26 (17.3)
	Community-based survey	5 (3.3)
	Randomized controlled trial	2 (1.3)
	Mixed study design	3 (2.0)
Geographical Region^a^	Asia	74 (49.3)
	Central and South America	30 (20.0)
	Africa	19 (12.7)
	Australo-Papua	14 (9.3)
	North America	11 (7.3)
	Europe	2 (1.3)
Year of publication	1978–1979	1 (0.6)
	1980–1989	8 (5.3)
	1990–1999	26 (17.3)
	2000–2009	39 (26.0)
	2010–2019	76 (50.7)
Language	English	141 (94.0)
	French	4 (2.7)
	Spanish	3 (2.0)
	Portuguese	2 (1.3)

^a^ Based on study site

### Spotting the BSN

Out of the total, twenty-three publications (15%) across 13 countries mentioned the number of victims that saw the BSN. In total, 2,723 victims out of 3,865 (70.5%) have seen the BSN (range 51.9% to 93.2% across studies) (Table 2). Factors associated with the circumstances/context of the bite were often described for those cases where the snake was not spotted. This includes, for example, bites that occurred at night, assumed to be caused by nocturnal snakes (e.g. *Trimeresurus sp*., *Bungarus sp*.) (e.g. Gabon, Hong Kong, Nepal, Saudi Arabia, Sri Lanka) [16–20] or in habitats with tall grass and thick plantation vegetation (e.g. Papua New Guinea, Nepal, central hills of Sri Lanka [21–23]) or dense rain forest vegetation (e.g. Ecuador [24]).

**Table 2:** Number of snakebite cases where the BSN was spotted by the victims.

Country	References	Number of publications	Total snakebite cases	Number BSNs spotted	BSN spotted (%)
Gabon	[16]	1	27	14	51.9
Nepal	[20, 22, 25]	3	149	78	52.3
Sri Lanka	[26–28]	3	1036	567	54.7
Papua New Guinea	[21]	1	205	117	57.1
Australia	[29–31]	3	345	218	63.2
Zimbabwe	[32]	1	84	54	64.3
Cameroon	[33]	1	57	37	64.9
Singapore	[34]	1	52	35	67.3
South Africa	[35, 36]	2	496	380	76.6
India	[37–40]	4	240	184	76.7
Colombia	[41]	1	485	414	85.4
Croatia	[42]	1	542	488	90.0
Greece	[43]	1	147	137	93.2
**Total/Average**		**23**	**3865**	**2723**	**70.5**

### BSN captured/killed and brought to health facilities

A total of 114 (76%) publications in 30 countries described snakebite cases where victims or bystanders captured the BSN and brought it to the health facility (S2 Table). In 78 (68%) of these 114 publications and 24 countries, the BSN was killed. Globally, the BSN was captured/killed in 9,671/25,188 (38%) snakebite cases, but this practice varied among countries (Table 3). Cultural perceptions affected this behaviour. In Nepal, an Indian cobra (*Naja naja)* was not killed by the victim “in fear of revenge by its partner” [20]. In Sri Lanka “there may be a reluctance to capture or kill the animal because of fear or superstition” [44], and a snakebite victim “refrained from catching the snake due to religious ethics” [27]. In South Africa (northern Natal), killed BSNs were often not brought to hospitals because “most snakes are incinerated immediately after having been killed for purposes of protection from the bones of the snake, which are believed to be dangerous even after death” [45].

**Table 3:** Geography and number of snakebite cases where the BSN was captured/killed by victims/bystanders.

Geographical region	Country	References	Number of publications ^a^	Total snakebite cases	Number BSNs captured	BSN captured %
Africa	Morocco	[46, 47]	2	905	14	1.5
	Tanzania	[48]	1	85	4	4.7
	Zimbabwe	[32]	1	250	22	8.8
	South Africa	[35, 45, 49, 50]	4	749	98	13.1
	Nigeria	[51, 52]	2	103	44	42.7
	Cameroon	[33, 53, 54]	3	118	60	50.8
Asia	Hong Kong	[17]	1	242	2	0.8
	Bangladesh	[55]	1	484	22	4.5
	Nepal	[20, 22, 25, 56]	4	898	74	8.2
	Taiwan	[57, 58]	2	71	8	11.3
	Sri Lanka	[23, 26, 28, 44, 59–67]	13	8 677	1 642	18.9
	Laos	[68, 69]	2	179	38	21.2
	Pakistan	[70]	1	90	21	23.3
	India	[37–40, 71–76]	10	775	189	24.4
	Saudi Arabia	[19, 77]	2	98	26	26.5
	Thailand	[78–82]	5	378	102	27.0
	Myanmar	[83, 84]	2	659	229	34.7
Australo-Papua	Papua New Guinea	[21]	1	335	10	3.0
	Australia	[29, 85–90]	7	667	181	27.1
Central and South America	Peru	[91]	1	170	10	5.9
	Ecuador	[92]	1	221	29	13.1
	Brazil	[93–106]	14	1 133	214	18.9
	Colombia	[41]	1	485	232	47.8
Europe	Croatia	[42]	1	542	49	9.0
	Greece	[43]	1	147	37	25.2
North America	USA	[107, 108]	2	176	27	15.3
	Puerto Rico	[109]	1	6	5	83.3
	**Total/Average**		**86**	**18 643**	**3 389**	**18.2**

^a^ This includes only publications where number of BSN captured/killed was available and excludes studies whose inclusion criteria required BSN to be brought to the hospital (13 publications) and countries with only one study reporting only a single snakebite case (i.e. case report) (2 publications).

In four publications, victims were bitten while attempting to kill a snake. This was the case for 53% and 100% of patients bitten on the fingers in two Australian studies [29, 30] and caused the death of an 80-year-old traditional healer in Cameroon [53] and a worker in Hong Kong [17].

### BSN identification methods

From the methods section of selected publications, we extracted information on the way the BSNs were identified when victims reached a healthcare facility (S2 Table). One or a combination of the following methods were used:

i) examining the captured BSN brought to the healthcare facility (110 publications). The person who identified the BSN was not always reported. In 43 publications, and particularly in Brazil (n = 10), Australia (n = 7), Sri Lanka (n = 7), and India (n = 6), the identification was made by a snake expert. This occurred particularly for case report/case series to confirm unusual snakebite events [85, 87, 94] and for randomized controlled trials of antivenoms or epidemiological studies focusing on a particular snake species. The latter involves patients bitten by a specific snake species and therefore giving precise taxonomic attribution to the biting snake is key.

ii) verbal description of the BSN by victims/bystanders (52 publications). They described the colour (e.g. brown or black) or size (e.g. large) of the BSN. In some studies, photographs or preserved specimens in curated collections of local material were shown to victims or bystanders to assist in the identification. Several publications mentioned that a majority of patients were unable to recognize snake species based on photographs (e.g. [35, 45, 66, 110]). Victims claimed to have recognized the BSN in 16 publications.

iii) clinical features (26 publications). Distinctive clinical syndromes associated with bites by individual species have been defined and algorithms developed to infer the BSN species in India and Sri Lanka [44, 76, 111]. In Brazil, health care providers identified the BSN at the genus level (*Bothrops/Crotalus/Lachesis/Micrurus*) based on patients’ signs and symptoms [102, 112, 113]. In these studies, most patients were effectively treated, but two deaths occurred possibly caused by the use of non-specific antivenom according to the authors [113]. The practice of bringing the BSN to the health facility is uncommon in northeastern Brazil compared to other Brazilian regions [104, 113]. Two studies focusing on krait bites also took into account bite circumstances (i.e. bite at night while victims were asleep).

iv) laboratory methods (23 publications). Immunoassays (EIA, ELISA) that detect venom antigens of some snake species in victims’ blood were used in Australia, Bangladesh, Brazil, Ecuador, Myanmar, Sri Lanka, and Thailand mainly to retrospectively identify BSNs and for research purposes. A pilot study explored the use of molecular tools to identify snakes in Nepal [56].

v) examining a picture of the BSN (9 publications). This practice was reported in the US (n = 3), Morocco (n = 2), Malaysia (n = 1), Australia (n = 1), Colombia (n = 1) and Laos (n = 1). The picture of the BSN was taken by the victim/bystanders, some with a mobile phone, or by the medical staff when the dead snake was brought to the hospital. The picture was then sent to a herpetologist (Laos, USA) or a Poison Control Center (Colombia, Malaysia, Morocco, USA).

BSNs were identified at the species or genus level in 18,065 out of 33,827 snakebite cases (53%) (S2 Table).

### Capability of victims and healthcare providers to identify snakes

#### Victims/bystanders

In nine studies, victims claimed they could identify the BSN and reported its common or local name (Table 4). In a Bangladeshi hospital, most snakebite victims were too distressed to describe the BSN and often misidentified it even when it was brought to the health facility [55]. Victims may not know the name of the snake [24] or the diversity of vernacular names given to a single snake species and/or taxonomic synonymies generate confusion for doctors (e.g. in the Brazilian Amazon [103, 112, 114]). In South Africa the word 'mamba' is often used by the local population to mean 'snake' of any species [50]. A survey of 150 inhabitants in southern Nepal showed that respondents were generally unable to identify different snake species [20]. In many parts of the world, authors considered snake identification by victims too unreliable to assist in routine snakebite management or in clinical snakebite research [24, 28, 66, 110].

**Table 4:** BSN identifications done by victims.

Country	References	Total BSNs seen	Victims who claimed they can identify the BSN Number (%)	Snake names reported by victims
Ecuador	[24]	142	43 (30)	Moash/Macanche, Equis, Sobrecama, Lora, Coral ^a^
Nepal	[22]	40	10 (25)	Cobra, krait, other venomous species not identified
Nepal	[25]	27	14 (52)	Cobra, krait, pit viper
Nepal	[115]	143^b^	87 (61)	Cobra, water snake, common krait
Nigeria	[116]	142	110 (77.5)	Cobra, carpet viper, black mamba, python
Nigeria	[52]	72	54 (75)	Carpet viper
South Africa	[45]	162	62 (38)	NR (53/62 identifications presumably correct)
South Africa	[36]	202	14 (6.3)	NR
Sri Lanka	[28]	206	158 (76.8)^c^	NR (104/158 accurate identifications)

^a^ Moash/Macanche (*Bothrops atrox*), Equis (*B*. *brazili*), Sobrecama (*B*. *asper*), Lora (*Bothrops bilineatus*), Coral (*Micrurus* species)

^b^ Includes all snakebite cases, not only cases where the snake was spotted

^c^ Identification based on dead snakes brought to health facility

NR: Not Reported

#### Healthcare providers

The identity and credentials of the person doing the identification of BSN brought to health facility was often not reported. This limits our assessment of healthcare provider capability in identifying snakes. In three studies in India, Sri Lanka, and Thailand, experts systematically re-examined dead snakes initially identified by hospital personnel and misidentifications were reported in respectively 17/44, 51/860, and 27/1631 cases [44, 117, 118]. In the US, pictures of the BSN were sent to a Poison Control Center and identification by a snake expert was compared to that of healthcare providers. Healthcare accuracy for copperhead (*Agkistrodon contortrix*) and cottonmouth (*A*. *piscivorus*) identifications was respectively 68% and 74% [119]. Many authors highlighted the lack of training of healthcare providers in identifying biting species including in Brazil, India, Nepal, Singapore, South Africa, and Thailand [34, 45, 56, 97, 113, 117, 118, 120, 121].

### BSN misidentifications

The BSNs were misidentified in 106 snakebite cases in Australia (n = 3), Brazil (n = 10), Hong Kong (n = 1), India (n = 6), Malaysia (n = 1), Sri Lanka (n = 54), and Thailand (n = 31). The consequences for the victims are detailed in Table 5 according to three scenarios. Most frequently, a venomous species was confused with another venomous species or a non or mildly venomous (opisthoglyphous) species was misidentified as a venomous snake.

**Table 5:** Cases of BSN misidentification published in the literature.

**Scenario 1: The BSN is venomous but misidentified as another venomous snake**							
**Country**	**References**	**Number of misidentfied BSNs**	**Initial identification (incorrect)**	**Person who did the incorrect identification^a^**	**Final identification (correct)**	**Person who did the correct identification^a^**	**Consequences for the victim(s)**
Australia	[30]	1	*Pseudonaja nuchalis*	Park ranger	*Notechis ater*	SVDK at hospital	Treated with Brown snake (*Pseudonaja sp*) antivenom and then Tiger snake (*Notechis sp*) antivenom. The 13-year old girl required prolonged ventilation and 53-day stay at hospital
Australia	[86]	1	*Pseudechis australis*	Other patients at hospital	*Pseudonaja nuchalis*	Flying Doctor at hospital	Treated with Black snake (*Pseudechis sp*) antivenom and then Brown snake (*Pseudonaja sp*) antivenom 7 hours after the bite. The patient died due to cerebral trauma (the patient had collapsed to the ground) aggravated by coagulation defect).
Brazil	[122]	1	Viperidae	NR	*Crotalus durissus terrificus*	Physician expert in toxicology	Treated with antibothropic serum and then intravenous crotalid antivenom. The patient remained with a severe behavioral and cognitive impairment.
Hong Kong	[17]	1	*Ophiophagus hannah*	Physician at hospital & Hong Kong Government Agriculture and Fisheries Department	*Naja naja*	NR	Below elbow amputation and death. Antivenom not administered due to uncertainty in snake species.
India	[40]	1	*Echis sochureki*	A colleague	*Macrovipera lebetina*	Experienced herpetologist	Inappropriate treatment with serum institute of India polyvalent^b^ that does not cover *M*. *Lebetina*, finger amputation
India	[117]	5	*Echis carinatus*	Hospital staff	*Hypnale hypnale*	Snake experts	Inappropriate treatment with Indian polyvalent antivenom that does not cover *H*. *Hypnale* (2/4 developed anaphylactoid antivenom reaction)
Sri Lanka	[59]	1	*Hypnale hypnale*	Native physician	*Daboia russelii*	NR, at hospital	Delayed treatment with indian polyvalent antivenom but patient died probably due to herniation of brain stem.
Sri Lanka	[44]	36	*Daboia russelii*	Hospital staff	*Hypnale hypnale*	Snake experts	Unnecessary use of antivenom
Thailand	[78]	8	*Bungarus fasciatus*	Medical staff	*Bungarus candidus*	Snake experts	Inappropriate treatment with *Bungarus fasciatus* antivenom. The patients died.
Thailand	[79]	1	*Naja kaouthia*	Assumption by the victim (in the dark)	*Bungarus fasciatus*	ELISA confirmation	Inappropriate treatment with monospecific cobra antivenom. The patient died.
Thailand	[118]	5	*Bungarus fasciatus*	Hospital staff	*Bungarus candidus*	Snake experts	Inappropriate treatment with *Bungarus fasciatus* antivenom
**Scenario 2: The BSN is non or mildly venomous (opisthoglyphous) but identified as a venomous snake**							
**Country**	**References**	**Number of misidentfied BSNs**	**Initial identification (incorrect)**	**Person who did the incorrect identification**^**a**^	**Final identification (correct)**	**Person who did the correct identification**^**a**^	**Consequences for the victim(s)**
Australia	[85]	1	Black snake	the family & SVDK	*Denisonia maculata*	Snake Experts	Inappropriate treatment with Australian Black Snake (*Pseudechis sp*) antivenom
Brazil	[123]	4	*Bothrops sp*.	Medical staff	*Tomodon dorsatus*	Laboratório de Herpetologia and the Laboratório Especial de Coleçóes Zoológicas of Butantan Institute.	Inappropriate treatment with Bothrops antivenom
Brazil	[97]	1	*Bothrops sp*.	Medical staff	*Philodryas patagoniensis*	NR	Inappropriate treatment with Bothrops antivenom
Brazil	[94]	1	*Bothrops sp*.	Medical staff / clinicial signs	*Clelia clelia plumbea*	Centra de Estudos e Pesquisas Biologicas at Universidade Catôlica de Goias	Inappropriate treatment with Bothrops antivenom
Brazil	[124]	1	*Bothrops sp*.	NR	*Drymarcon corais*	NR	Inappropriate treatment with Bothrops antivenom, nearly died of severe anaphylaxis
Brazil	[124]	1	*Bothrops sp*.	NR	*Sibynomorphus mikànii*	NR	Inappropriate treatment with Bothrops antivenom, no side effect
Malaysia	[125]	1	*Bungarus Sp*.	National Poison Centre / description of a photo of the snake	*Chrysopelea pelias*	Snake Experts	Inappropriate treatment with Indian polyvalent antivenom
Sri Lanka	[64]	1	*Echis carinatus*	Hospital staff	*Boiga trigonata*	Snake expert / Faculty of Medicine, Peradeniya	NR
Sri Lanka	[44]	2	*Hypnale hypnale*	Hospital staff	*Boiga ceylonensis/B*. *trigonata*	Snake experts	Unnecessary use of antivenom
Sri Lanka	[44]	3	*Daboia russelii*	Hospital staff	*Boiga ceylonensis/B*. *trigonata*	Snake experts	Unnecessary use of antivenom
Sri Lanka	[44]	1	*Echis carinatus*	Hospital staff	*Boiga ceylonensis/B*. *trigonata*	Snake experts	Unnecessary use of antivenom
Sri Lanka	[44]	2	*Echis carinatus*	Hospital staff	Pythons	Snake experts	Unnecessary use of antivenom
Sri Lanka	[44]	5	*Bungarus caeruleus*	Hospital staff	*Lycodon aulicus/L*. *striatus sinhalayu*	Snake experts	Unnecessary use of antivenom
Sri Lanka	[44]	2	Cobras	Hospital staff	Rat snakes	Snake experts	Unnecessary use of antivenom
Thailand	[118]	2	*Calloselasma rhodostoma*	Hospital staff	*Oligodon dorsolateralis*	Snake experts	Unnecessary use of antivenom
Thailand	[118]	1	*Daboia russelii*	Hospital staff	*Oligodon cyclurus*	Snake experts	Unnecessary use of antivenom
Thailand	[118]	1	*Calloselasma rhodostoma*	Hospital staff	*Oligodon cyclurus*	Snake experts	Unnecessary use of antivenom
Thailand	[118]	7	*Daboia russelii*	Hospital staff	*Boiga multomaculata*	Snake experts	Unnecessary use of antivenom
Thailand	[118]	1	*Bungarus candidus*	Hospital staff	*Lycodon luoensis*	Snake experts	Unnecessary use of antivenom
Thailand	[118]	1	*Calloselasma rhodostoma*	Hospital staff	*Rhabdophis subminiatus*	Snake experts	Unnecessary use of antivenom
Thailand	[118]	4	*B*. *fasciatus/B*. *candidus*	Hospital staff	*Dryocalamus davisonii*	Snake experts	Unnecessary use of antivenom
**Scenario 3: The BSN is venomous but identified as non-venomous**							
**Country**	**References**	**Number of misidentfied BSNs**	**Initial identification (incorrect)**	**Person who did the incorrect identification**^**a**^	**Final identification (correct)**	**Person who did the correct identification**^**a**^	**Consequences for the victim(s)**
Brazil	[98]	1	Nonpoisonous	Healthcare provider	*Bothrops jararacussu*	NR	Delayed treatment with anti-Bothrops serum, intracranial bleeding and gradual improvement
Sri Lanka	[60]	1	*Lycodon aulicus*	Doctor	*Bungarus caeruleus*	Identified by expert after patient death	Delayed administration of Indian polyvalent anti-venom, death 46 hours after the bite

^a^ Identification done by examining the snake unless otherwise specified

^b^ Indian polyvalent antivenom is raised against the venom of *B*. *caeruleus*, *Daboia russelii*, *Echis carinatus*, and *N*. *naja* (the so-called Big Four).

NR: Not Reported

### Diversity of captured/killed BSN

A total of 8,885 BSNs from 149 species in 12 families were captured and identified. Of these, 6,750 BSNs were identified to species, 2,082 to genus, and 53 as “non-venomous”. In total, 7,628 individual snakes of 69 species were MIVS, 1,205 individuals of 71 species were non-venomous, and 52 individuals of 9 species were potentially dangerous but understudied species (species that lack specific data on clinical symptoms or venom toxicity but which are closely related to dangerous snakes and possess fangs and venom glands (see [126]) (Table 6 and S3 Table). The 149 species included 40 species in the family Viperidae, 35 species in the family Elapidae, 49 species in the family Colubridae, 4 species in the family Lamprophiidae, and 21 species from 8 other families (Acrochordidae, Boidae, Cylindrophiidae, Homalopsidae, Pareidae, Pythonidae, Typhlopidae, Xenopeltidae; S3 Table), as well as one amphisbaenid lizard (*Amphisbaena mertensii)[101]* and one serpentine amphibian (caecilian [118]) (not included in snake totals above). Of these 149 snake species, 32 were diurnal, 76 were nocturnal, and 41 were or could be active throughout the day, depending on the season. A total of 84 species were active foragers, 45 were ambush predators, 14 used both strategies, and 6 were unknown. General habitat use of 26 species was arboreal, 7 fossorial, 22 aquatic or semi-aquatic, and 94 were primarily terrestrial, with 4 of these partially aquatic, 5 partially arboreal, and 7 partially fossorial (S4 Table).

**Table 6:** Diversity and abundance of BSNs captured/killed and identified.

Geographical region	MIVS (n)	Non-venomous snakes (n)	Potentially dangerous snakes (n)
Africa	*Atractaspis bibronii (22)*	*Afrotyphlops schlegelii (1)*	*Causus defilippi (8)*
	*Bitis arietans (8)*	*Boaedon capensis (2)*	*Causus rhombeatus (9)*
	*Bitis atropos (1)*	*Crotaphopeltis hotamboeia (3)*	* *
	*Daboia mauritanica (3)*	*Dasypeltis scabra (1)*	* *
	*Dendroaspis polylepis (12)*	*Philothamnus spp*. *(2)*	* *
	*Echis romani (103)*	* *	* *
	*Hemachatus haemachatus (1)*	* *	* *
	*Naja annulifera (3)*	* *	* *
	*Naja melanoleuca (1)*	* *	* *
	*Naja mossambica (18)*	* *	* *
Asia	*Bitis arietans (1)*	*Acrochordus javanicus (NR)*	* *
	*Bungarus caeruleus (623)*	*Ahaetulla nasuta (10)*	* *
	*Bungarus candidus (17)*	*Ahaetulla prasina (NR)*	* *
	*Bungarus fasciatus (6)*	*Argyrogena fasciolata (1)*	* *
	*Bungarus lividus (3)*	*Atreitum schistosum (1)*	* *
	*Bungarus multicinctus (6)*	*Boiga ceylonensis (10)*	* *
	*Bungarus niger (1)*	*Boiga cyanea (NR)*	* *
	*Bungarus spp*. *(20)*	*Boiga cynodon (NR)*	* *
	*Calloselasma rhodostoma (448)*	*Boiga forstenii (8)*	* *
	*Cerastes cerastes (7)*	*Boiga multomaculata (NR)*	* *
	*Cerastes gasparetti (7)*	*Boiga sp*. *(4)*	* *
	*Cerastes vipera (1)*	*Boiga trigonata (4)*	* *
	*Daboia russelii (985)*	*Chrysopelea ornata (1)*	* *
	*Daboia siamensis (383)*	*Chrysopelea pelias (1)*	* *
	*Echis carinatus (229)*	*Coelognathus helena (5)*	* *
	*Echis coloratus (5)*	*Coelognathus radiatus (4)*	* *
	*Hydrophis cyanocinctus (2)*	*Cylindrophis maculatus (1)*	* *
	*Hydrophis platurus (2)*	*Cylindrophis ruffus (NR)*	* *
	*Hydrophis schistosus (2)*	*Enhydris enhydris (NR)*	* *
	*Hydrophis spiralis (1)*	*Enhydris jagorii (NR)*	* *
	*Hypnale hypnale (502)*	*Gonyosoma oxycephalum (NR)*	* *
	*Hypnale nepa (5)*	*Homolopsis buccata (NR)*	* *
	*Hypnale spp*. *(1930)*	*Hypsiscopus plumbea (NR)*	* *
	*Hypnale zara (17)*	*Indotyphlops braminus (NR)*	* *
	*Macrovipera lebetina (1)*	*Lycodon aulicus (57)*	* *
	*Naja atra (1)*	*Lycodon capucinus (NR)*	* *
	*Naja kaouthia (108)*	*Lycodon davisonii (NR)*	* *
	*Naja naja (328)*	*Lycodon laoensis (NR)*	* *
	*Naja siamensis (118)*	*Lycodon spp*. *(3)*	* *
	*Naja spp*. *(8)*	*Lycodon striatus (15)*	* *
	*Naja sumatrana (1)*	*Malpolon moilensis (4)*	* *
	*Ovophis monticola (1)*	*« non-venomous snakes » (53)*	* *
	*Rhabdophis subminiatus (5)*	*Oligodon arnensis (7)*	* *
	*Trimeresurus albolabris (344)*	*Oligodon cyclurus (NR)*	* *
	*Trimeresurus gramineus (20)*	*Oligodon spp (7)*	* *
	*Trimeresurus kanburiensis (1)*	*Oligodon taeniatus (NR)*	* *
	*Trimeresurus macrops (23)*	*Oligodon taeniolatus (2)*	* *
	*Trimeresurus malabaricus (26)*	*Pareas carinatus (NR)*	* *
	*Trimeresurus purpureomaculatus (3)*	*Psammodynastes pulverulentus (2)*	* *
	*Trimeresurus spp*. *(36)*	*Ptyas mucosa (13)*	* *
	*Trimeresurus trigonocephalus (19)*	*Ptyas spp*. *(2)*	* *
	*Tropidolaemus wagleri (2)*	*Python molurus (2)*	* *
	*Walterinnesia aegyptia (1)*	*Subsessor bocourti (NR)*	* *
	* *	*Xenochrophis asperrimus (2)*	* *
	* *	*Xenochrophis flavipunctatus (1)*	* *
	* *	*Xenochrophis flavipunctatus (NR)*	* *
	* *	*Xenochrophis piscator (12)*	* *
	* *	*Xenochrophis spp*. *(2)*	* *
	* *	*Xenopeltis unicolor (NR)*	* *
Australo-papua	*Acanthophis sp*. *(13)*	*Antaresia childreni (12)*	*Boiga irregularis (3)*
	*Notechis scutatus (NR)*	*Dendrelaphis punctulatus (2)*	*Cryptophis pallidiceps (4)*
	*Oxyuranus microlepidotus (1)*	*Fordonia leucobalia (1)*	*Demansia olivacea (5)*
	*Oxyuranus scutellatus (1)*	*Liasis fuscus (9)*	*Demansia spp*. *(17)*
	*Pseudechis australis (30)*	*Liasis olivaceus (4)*	*Denisonia maculata (1)*
	*Pseudechis guttatus (1)*	*Morelia spilota (8)*	*Furina ornata (3)*
	*Pseudonaja nuchalis (13)*	*Pseudoferania polylepis (1)*	*Hemiaspis signata (2)*
	*Pseudonaja spp*. *(20)*	*Stegonotus australis (10)*	* *
	* *	*Tropidonophis mairii (2)*	* *
Central and South America	*Bothrops alternatus (6)*	*Apostolepis assimilis (1)*	* *
	*Bothrops atrox (45)*	*Boa constrictor (2)*	* *
	*Bothrops bilineatus (12)*	*Clelia plumbea (1)*	* *
	*Bothrops erythromelas (35)*	*Dipsas mikanii (6)*	* *
	*Bothrops jararaca (779)*	*Drymarchon corais (1)*	* *
	*Bothrops jararacussu (1)*	*Erythrolamprus poecilogyrus (2)*	* *
	*Bothrops moojeni (70)*	*Erythrolamprus spp*. *(1)*	* *
	*Bothrops neuwiedi (30)*	*Eunectes murinus (1)*	* *
	*Bothrops sp*. *(15)*	*Oxyrhopus trigeminus (3)*	* *
	*Bothrops taeniatus (2)*	*Palusophis bifossatus (3)*	* *
	*Crotalus durissus (14)*	*Philodryas mattogrossensis (1)*	* *
	*Micrurus frontalis (2)*	*Philodryas olfersii (4)*	* *
	*Micrurus lemniscatus (2)*	*Philodryas patagoniensis (300)*	* *
	*Micrurus spp*. *(2)*	*Simophis rhinostoma (2)*	* *
	* *	*Tomodon dorsatus (86)*	* *
	* *	*Xenodon merremii (3)*	* *
Europe	*Vipera ammodytes (83)*	* *	* *
	*Vipera berus (3)*	* *	* *
North America	*Agkistrodon contortrix (26)*	*Borikenophis portoricensis (5)*	* *
	*Crotalus cerastes (1)*	* *	* *

In this table, snake scientific names follow the taxonomy of the Reptile Database as of October, 2019 [10]

NR: Not reported

Within a country, the total number of snake bites across all publications was positively correlated with the number of species of BSNs reported (Panel A in S1 Fig; PPC = 0.55, p < 0.001), especially among non-venomous snakes, whereas the number of snake species per country was more weakly correlated with the number of species of BSNs reported (Panel B in S1 Fig; PPC = 0.36, p = 0.04).

A substantial number of snakes—1,859 (21%) individuals of 22 species—were reported in the literature using only the common name. Of those reported using the scientific name, 20 species (92 individuals) had been moved to another genus since the time of publication, 10 species (241 individuals) had experienced other taxonomic changes (splits or lumps) (e.g. *Echis ocellatus* in Cameroon has been renamed *E*. *romani* [127]), and the names of 3 species (6 individuals) contained minor misspellings as reported (S3 Table).

## Discussion

Based on a final selection of 150 publications from across the world, this first scoping review on BSN identification practices of communities and healthcare providers confronted with snakebite shows that: (I) BSN identification is important for snakebite epidemiology and clinical management yet there is a diversity of practices depending on cultural, ecological and healthcare contexts and there are no official standards (II) the majority of victims see the BSN but they are unreliable in their identification (III) the practice of capturing or killing the BSN occurs in all continents, especially in Asian countries with a diversity of snakes, and (IV) healthcare providers struggle to identify a BSN presented in the health facility and misidentifications occur.

Snakebite victims/bystanders in many snakebite endemic countries are aware of the importance of BSN identification and can play an important role. We found that on average 70% of victims/bystanders spotted the BSN and 38% managed to capture or kill it and brought it to the health facility for identification. Although this is a dangerous practice (i.e. due to the risk of secondary bites [17, 29, 30, 53, 128–133]), it occurs worldwide but particularly in Asia (e.g. Myanmar, India, and Sri Lanka). Communities in these snakebite endemic countries believe that bringing the BSN to the health facility for identification is essential for treating the victim [11, 134]. Myths and beliefs regarding snakes are present in many societies (e.g. [135, 136]) and could prevent victims/bystanders from killing the snake, yet the influence of these cultural aspects on BSN identification was rarely addressed in the selected publications and would deserve more research. Traditional healers may have a good knowledge on the type of snakes in their local environment, their distribution and behaviours. Yet, in this review, we did not find articles that report their role in BSN identification. Further studies could explore if their snakebite remedies are species-specific and assess their knowledge of local snake diversity. Overall, traditional healers can act as partners with healthcare providers promoting prompt referral to health facilities in case of snake bites inflicted by venomous snakes.

Photographing the BSN (e.g. with mobile phones) is an emerging practice in snakebite endemic countries worldwide. This was described for Australia, Colombia, Laos, Malaysia, Morocco, and the US, where snake experts are involved in snake photo identification (e.g. at Poison Control Center). This procedure is recommended by the WHO [12], the Snakebite Healing and Education Society in India [137] and the African Snakebite Institute [138] among others and it is certainly less dangerous than killing or capturing the snake. Even fast-moving, active-foraging snakes (e.g. *Demansia*, *Dendroaspis*, *Naja*) and secretive, fossorial species (e.g. *Atractaspis*, *Micrurus*, *Xenopeltis*) were captured/killed in our review and could have been photographed instead. The advantages of using photos for BSN identification are numerous. Firstly, photos are less subject to interpretations and thus more reliable than victim descriptions. We showed that victims in general described the BSN colour or size, which is of limited value for the identification, and their identification of the BSN is often unreliable even with the assistance of preserved specimens or local snake photographs. Secondly, photos can be rapidly shared between victims, healthcare providers, and snake biologists, accelerating the identification process and improving its accuracy. Snake identification services based on snake photos have been developed in India [139], Sri Lanka [140], or Thailand [141] and are provided by poison control centres in the US [142] and Colombia [143]. This could be developed in other countries and, in low-resource settings, snake identification could be even crowdsourced involving online communities of snake experts (e.g. open biodiversity platforms like HerpMapper, iNaturalist, or snake identification Facebook groups) [144, 145]. Thirdly, photos can be securely and indefinitely stored building a digital dataset. Besides the ecological and epidemiological value of such a dataset, it could serve to train healthcare providers and machine learning algorithms in snake classification [146, 147].

BSN identification is particularly important in certain SBE endemic regions where monovalent or bi-/tri valent antivenoms are the only affordable treatment. This is the case, for example, in Myanmar, Taiwan, and Thailand in East and South East Asia, but also in many sub-Saharan Africa regions where *Echis* snake bites are prevalent [12]. Besides this, BSN identification is important for ecological and epidemiological purposes and subsequently for an optimal antivenom coverage [146] (e.g. Hump-nosed viper (*Hypnale hypnale*) causes frequent bites with morbidity and mortality in Sri Lanka and southwest India but currently lacks effective antivenom) [117, 148]. Healthcare providers should be encouraged to photograph and archive images of BSN brought to health facilities to help build national or regional BSN photo repositories for further epidemiological studies.

BSN identification could also complement syndromic approach to snakebite [36, 44, 76], particularly in those cases where symptoms caused by venoms of different snake species overlap (e.g. Russell’s viper causes paralytic signs suggesting elapid neurotoxicity in Sri Lanka) [12].

Snake species diversity and the fact that bites from some “non-venomous” snakes can cause signs of envenoming (e.g. *Clelia plumbea* and *Philodryas olfersii* in Brazil) [64, 94, 124, 126] complicate snakebite clinical management and have led to snake misidentifications. Snake identification can be challenging for communities and healthcare providers (i.e. diversity of snakes, mimicry). In two villages in rural Tanzania, most of the respondents to a survey could not precisely differentiate between venomous and non-venomous snakes [149]. In a cross-sectional survey of 119 healthcare providers in Laos, 86 participants (72.3%) had inadequate knowledge of snake identification [150]. Although healthcare providers could be trained to recognize locally prevalent snake species (e.g. at the Damak Snakebite Treatment Center in Nepal [151]), reinforcing the collaboration between communities at risk, healthcare providers and snake experts could be a more effective approach to increase the number of BSNs correctly identified.

With this review, we retrospectively collated all the species names of the 8,885 BSN that were captured and identified and we have built a first extensive global list of snakes having bitten humans. This list includes 69 MIVS and 71 non-venomous snake species, as well as nine that are potentially dangerous but understudied species [126]. MIVS species are already listed by the WHO (2010) [152]. This review extends this list to non-venomous snakes, although there is significant bias in which non-venomous snakes are brought to health facilities and the number of non-venomous BSN species was strongly correlated with the number of snake bites, suggesting that many more species of non-venomous BSNs exist and a limit has not been reached within epidemiological data. Nevertheless, this confirms that many snake bites globally are caused by non-venomous snakes, although victims may not always realize this immediately or at all.

## Limitations of this study

Many publications had to be excluded from the review because the way the BSN identification was done was unclear or not reported even though the BSN genus or species was mentioned. The list of BSN species we built is limited to the publications we retrieved and included in the review. It could have been extended, had more publications met the eligibility criteria. We recommend that future epidemiological studies on snakebite clearly describe the method(s) used to identify the BSN, including the credentials of the person who did the identification and their confidence in the identification. We used key words related to ‘identification’ to specifically gather snakebite publications describing snake identification, although we may have missed relevant publications that do not mention these keywords or that are published in other languages (e.g. Russian, Chinese). Information on snake identification was reported in an inconsistent and fragmented manner. We managed this problem by involving two authors in data extraction and comparing data collected until a consensus was reached. Some publications, particularly prospective studies, applied specific inclusion criteria (e.g. a specific snake species, dead snake brought to hospital). These were excluded from analyses where they were sources of bias (e.g. calculation of proportion of captured snakes). The selected publications are mainly hospital-based studies with very few community-based studies. We missed the behaviour of snakebite victims who did not seek treatment at hospitals because of asymptomatic bite or use of traditional healers. Snake taxonomy is constantly changing and an average of 30 new species per year have been described since the year 2000. Although we were always able to definitively decide which species/genera were meant, rare or newly-described species may be missed by all identification methodologies, and taxonomic instability further complicates an already-challenging situation [153, 154]. Finally, we cannot account for situations where snake misidentification was never discovered and incorrect names have been published, which seems likely in a subset of cases.

## Conclusion

This global scoping review showed that BSN identification in snakebite endemic countries includes a diversity of methods and practices: capturing/killing the BSN and examination of its body, description of the BSN by victim/bystanders, interpretation of clinical features, laboratory tests, and photographing the BSN. The capacity of snakebite victims, bystanders and healthcare providers to spot and identify the BSN is context-specific and depends on circumstances of the bite, the local snake diversity, and their own knowledge of local snakes. BSN misidentifications occur and lead to inappropriate management of the victims. The influence of cultural perceptions about snakes and role of traditional healers in snake identification are largely unexplored in the literature and urges for further research. Victims/bystanders managed to capture a diversity of BSNs, including fast-moving nervous snakes. This is dangerous and not recommended, and photographing the snake could be an alternative option [12]. We provided the first evidence-based list of venomous and non-venomous snake species involved in bites to humans. This list could be further extended by implementing snake identification as part of the clinical practice. Such a systematic collection of the taxonomy of BSNs at the global level is of considerable interest to better understand snake ecology and snakebite epidemiology and ultimately improve SBE management.

## Supporting information

S1 Table Search strategy syntax for each bibliographic database. (DOCX)

S2 Table Dataset—Scoping review identification. (XLSX)

S3 Table Taxonomic identification of captured biting snakes. (XLSX)

S4 Table Behaviour and ecology of captured biting snakes. (XLSX)

S1 Fig Correlation between number of snake bites and occurring snake species with number of biting snake species. Correlations between A) the total number of snake bites across all publications and B) the total number of snake species occurring in a country with the number of species of BSNs reported. Each dot represents a country. Thailand is missing from the non-venomous panel in part A because quantitative data are not given for non-venomous BSNs in [31]. MIVS = medically-important venomous snakes.
(TIFF)

S1 File PRISMA-ScR checklist. (PDF)
