# Supplementary material for: Identifying the snake: First scoping review on practices of communities and healthcare providers confronted with snakebite across the world
Source: PLoS One. 2020 Mar 5;15(3):e0229989. doi: 10.1371/journal.pone.0229989 (PMC7058330; doi:10.1371/journal.pone.0229989)
Supplement: S1 Table — (DOCX) [file pone.0229989.s001.docx]

**S1 Table. Search strategy syntax for each bibliographic database**

| **Database** | **Syntax Combination** |
| --- | --- |
| **PubMed**  **Search Field**  **[All Fields]** | (Snakebite* OR “snake bite*” OR (snake AND envenoming) OR (snake AND envenomation)) AND (case OR cases OR victims OR victim OR event OR events OR patient OR patients) AND (“biting snake” OR “biting snakes” OR culprit* OR "offending snake" OR “offending snakes” OR species) AND (identif* OR misidentif* OR unidentif* OR identity OR mistaking)    A filter on species (Human) was applied to search results |
| **Web of Science**  **Search Field**  **[Topic]** | (Snakebite* OR “snake bite*” OR (snake AND envenoming) OR (snake AND envenomation)) AND (case OR cases OR victims OR victim OR event OR events OR patient OR patients) AND (“biting snake” OR “biting snakes” OR culprit* OR "offending snake" OR “offending snakes” OR species) AND (identif* OR misidentif* OR unidentif* OR identity OR mistaking) |
