# Supplementary figures and images for: Identifying the snake: First scoping review on practices of communities and healthcare providers confronted with snakebite across the world
Source: PLoS One. 2020 Mar 5;15(3):e0229989. doi: 10.1371/journal.pone.0229989 (PMC7058330; doi:10.1371/journal.pone.0229989)

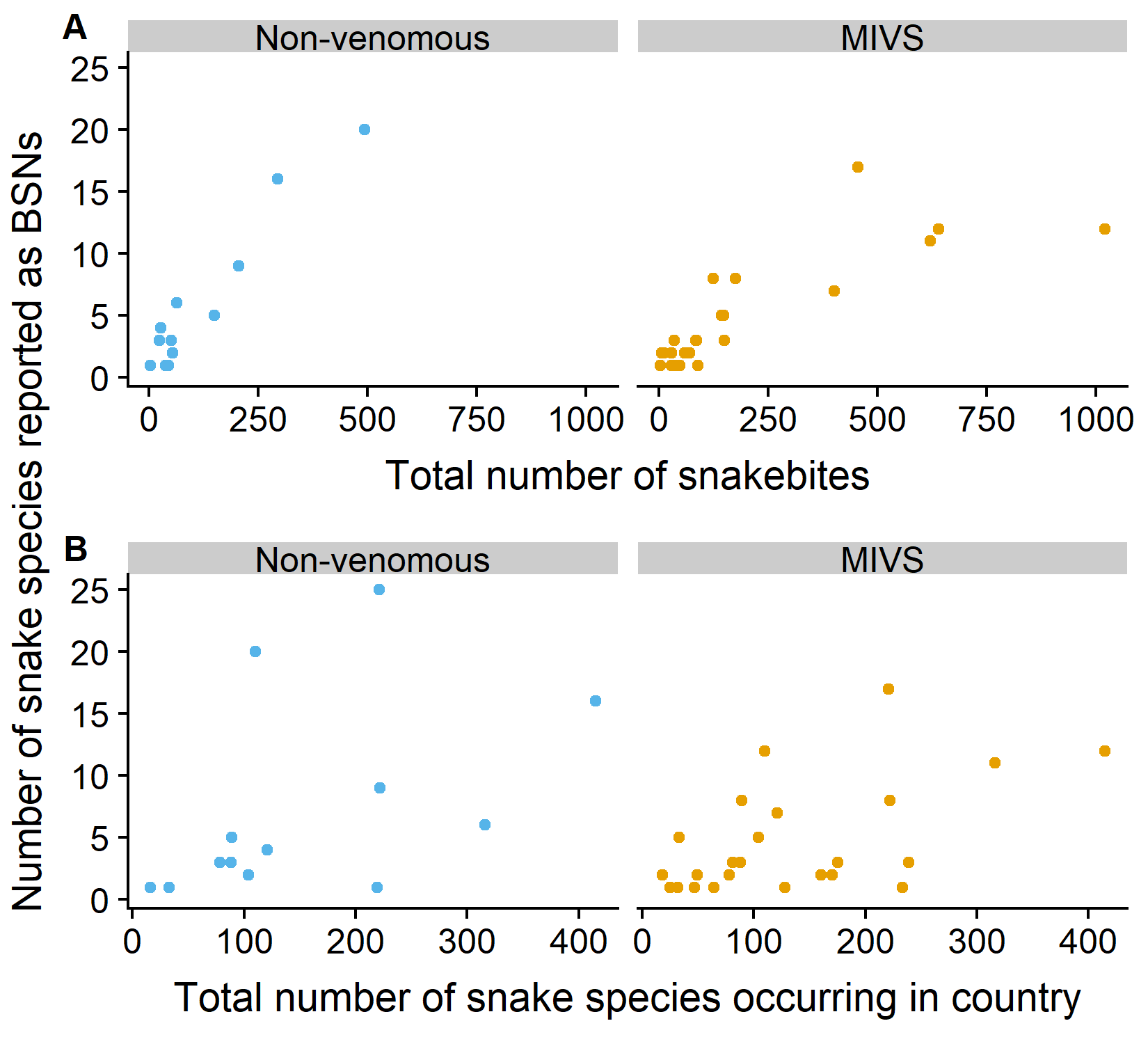

Supplement: S1 Fig — Correlations between A) the total number of snake bites across all publications and B) the total number of snake species occurring in a country with the number of species of BSNs reported. Each dot represents a country. Thailand is missing from the non-venomous panel in part A because quantitative data are not given for non-venomous BSNs in [31]. MIVS = medically-important venomous snakes. (TIFF) [file pone.0229989.s005.tiff]
